# Medical work assessment in German hospitals: a study protocol of a movement sequence analysis (MAGRO-MSA)

**DOI:** 10.1186/s12995-014-0040-7

**Published:** 2015-01-09

**Authors:** Daniela Ohlendorf, Mario Schwarzer, Julia Rey, Ingo Hermanns, Albert Nienhaus, Rolf Ellegast, Dirk Ditchen, Stefanie Mache, David A Groneberg

**Affiliations:** Institute of Occupational Medicine, Social Medicine and Environmental Medicine, Goethe-University Frankfurt/Main, Theodor-Stern-Kai 7, Building 9a, Frankfurt/Main, 60596 Germany; Institute of Biostatistics and Mathematical Modeling, Goethe-University, Frankfurt/Main, Germany; Institute for Occupational Health and Safety (IFA) of the German Social Accident Insurance (DGUV), Sankt Augustin, Germany; Principles of Prevention and Rehabilitation Department (GPR), Institute for Statutory Accident Insurance and Prevention in the Health and Welfare Services (BGW), Hamburg, Germany

**Keywords:** Body posture, CUELA system, Movement sequence, Physicians’ health, Range of motions, Working task

## Abstract

**Background:**

Medical doctors are essential for the German public and occupational health system. They ensure the productivity of German society by enabling people to regain and recover their health. That is why the physicians’ health and hence their productivity require special attention. Musculoskeletal disorders have a high prevalence in this work area. As a consequence, movement sequences, range of motions, and body postures of physicians in the course of the working day are in focus of this research project.

**Methods:**

For this investigation 21 male or female junior physicians of various conservative medical disciplines will be covered. Data will be collected over one working day (approx. 9 hours). The CUELA system attached to the test person’s body detects body posture and/or movements. This biomechanical measurement system ensures a locomotor and posture analysis that includes movement sequences, movement intensity, and range of motions to qualify the work tasks. For data analysis intra- and inter-professional comparisons are chosen.

**Conclusions:**

Working movement sequence analysis of physicians by means of the CUELA system is exclusive and novel in its focus. Up to now, knowledge of the working tasks of medical doctors has only been acquired by real-time observation approaches to work activity. In addition to this method of analysis, the CUELA system is able to record quantified biomechanical data about musculoskeletal loads of ordinary working tasks. Workloads and activities of physicians can be improved by ergonomic work design to reduce musculoskeletal disorders by utilizing the data collected. The healthcare system in Germany will thus be optimized by improving medical doctors’ health. Consequently, MAGRO-MSA will also be used for other healthcare professions such as nurses and physicians assistants.

## Background

The healthcare professions are commonly in the focus of occupational medicine research since they involve numerous potential hazards [1-4].

In the physician’s profession the principal focus is on the patient, and his or her health is the main priority. However, the physician’s health and physical exertion are mostly neglected.

One of the first approaches was real-time observation that provided an initial insight into the routine of the working day or the complexity of occupational activities in various disciplines of physicians such as pediatricians, gastroenterologists, geriatricians, psychiatrists, or specialists in internal medicine [5-14]. Individual work steps and work requirements were precisely recorded in time and the daily workload was analyzed by means of a temporary contribution percentage. In this context the main activities were divided into two categories: direct and indirect patient contact. These two categories were divided into sub-categories to provide a more comprehensive view. Indirect patient contact activities consist mainly of medical reports and further documentation.

Due to the complexity of medical occupations the profession has to be permanently physically active throughout its working day [15]. In this context a variety of activities over a shorter or even a longer time period must be performed. In this connection ergonomic movements are often impractical due to the particular time or spatial circumstances, hence, awkward postures are required. High physical workloads impact on this professional group and can potentially lead to postural physical discomfort and muscular imbalance. An imbalance between the external loads and the load capacity of the active and passive movement system leads to physical complaints. Repeated movement stereotypes cause neuromuscular imbalances and may lead to an intensification of the malposture [16].

### Objectives

A biomechanical measurement system for locomotor and posture analysis is required to evaluate the workflow. In this case measurement techniques are able to analyze movements and body postures more objectively than survey and questionnaire methods [17].

The general purpose of a measurement technique-based job task analysis is to document the range of motion and the movement intensity while fulfilling daily work tasks such as by using the CUELA system (German abbreviation for “computer-assisted recording and long-term analysis of musculoskeletal loads”). The ambulatory CUELA system was developed by the Institute for Occupational Safety and Health of the German Social Accident Insurance in St. Augustin, Germany [18,19]. It can continuously record body postures and movements.

The system can capture every single activity undertaken in a shift (a period of nine hours). An optional video camera was used to record all job tasks in addition to the CUELA system. This helps to gain an overview of all movements and reduces or even eliminates movement artefacts that could not be registered by the CUELA system alone. Past studies have used the CUELA system successfully on, for example, flight attendants [20], occupational drivers [21], animal facility washroom employees [22], or nurses [23]. It has also been utilized in different professions in order to analyze knee-straining activities [24].

Most of the movement analysis techniques, such as the Vicon system, focus on registration of kinetic and kinematic data during the performance of certain tasks under laboratory conditions [25]. These techniques ensure a high measurement accuracy but provide only a semi-realistic report of work activities because they measure them under laboratory conditions and the subjects are not able to leave the laboratory. A real-time transfer between these variables and the adequate movement may be hindered.

The procedure described here tries to draw parallels between real-time job task descriptions as already known and specifying the activities conducted [5-14] and the further question of how these activities are physically performed. The CUELA system is a person centered posture measuring system to enable a true-to-nature reproduction of work activities in the original work environment because it is attached to an individual and detects all data during real-life working activities. Simulations of working conditions thus become obsolete and test persons can perform their ordinary tasks.

The present occupational health investigation creates working profiles in terms of biomechanical parameters of the external stress and load degree of the locomotor system such as angles of the cervical, thoracic, and lumbar spine area as well as arm and leg angles. Based on this framework a real-time insight into the work tasks enables a closer analysis of the work routine.

Consequently, the aim of the present study is to evaluate the workload areas of conservative (non-surgical) junior physicians. The investigation focuses on identifying effective and ineffective work movements in one activity or in several activities or activity sequences. For this purpose a multimodal movement measurement approach to musculoskeletal loads evaluates workload and task intensity by recording the objective workload execution. Furthermore, it is necessary to identify the range of motion and the movement intensity. This analysis is helpful for effectively reducing complaints and disorders of the posture and movement apparatus in the long term [26]. Based on these investigation results the same analyses are needed for physicians’ assistants to address their working profile as well. Only when physicians and their assistants can reduce their discomfort as a result of these analyzes can the German healthcare system benefit [27].

#### Posture evaluation

In general, a human being can shift in space on three planes: (1) forward/backward (x direction), (2) sidewise (right/left) (y direction) and (3) upwards and downwards (z direction), so that a body is able to move in a sagittal (anterior/posterior), frontal (medial/lateral), or transverse plane (horizontal; vertical along the longitudinal axis) [28]. Ergonomic postures are defined in the ISO 11226 [29] and DIN EN 1005–4 [30] standards, especially for the trunk postures.

These standards correspond to an upright trunk posture if there are inclinations of between 0° and 20°. According to DIN EN1005-1 [31] standards, trunk postures are static postures when they are maintained constantly for 4 seconds under a slightly changing force.

**Figure 1:**
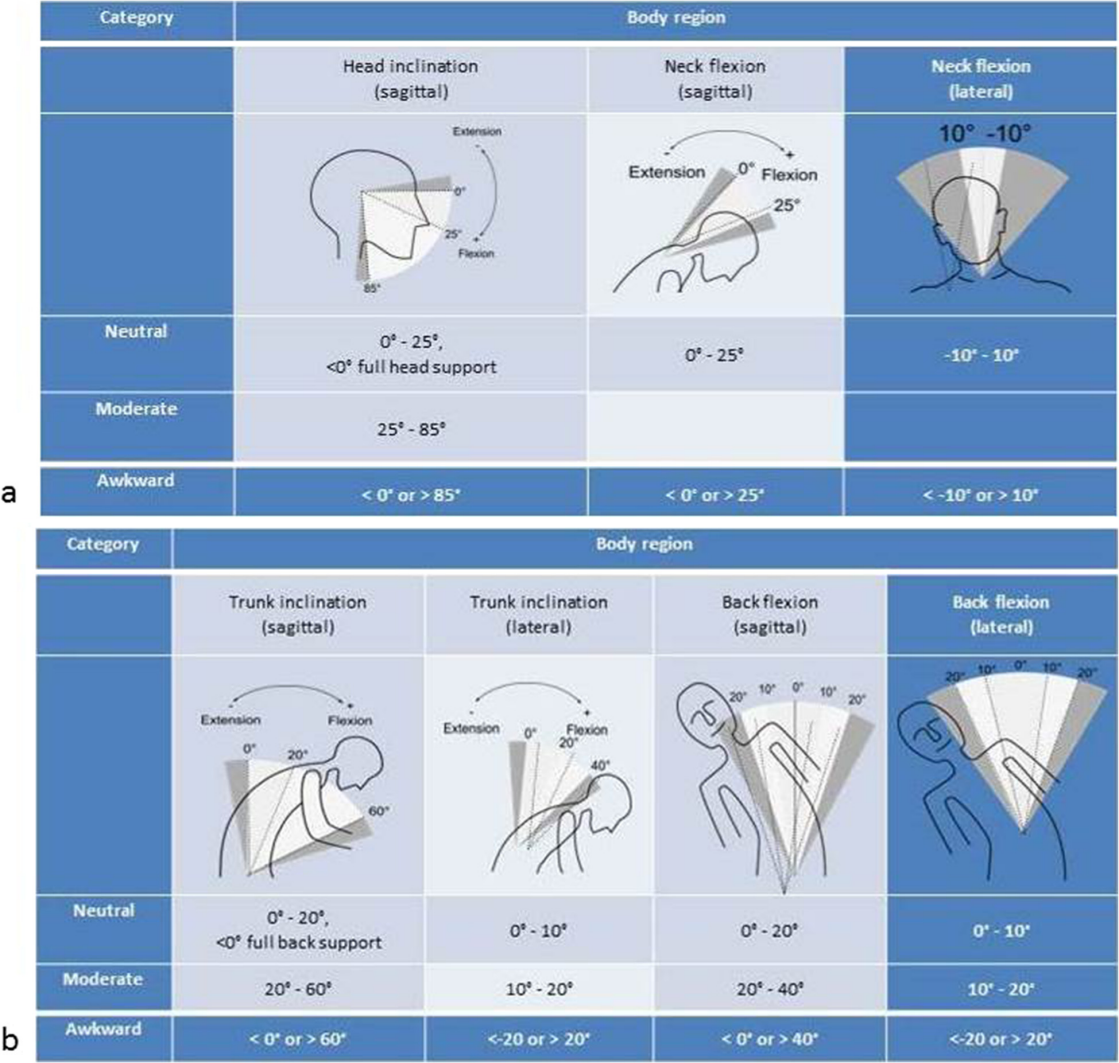
**Classification of body posture. a**. Classifications of head inclination (sagittal plane) and neck flexion (sagittal and lateral plane) in three categories: neutral, moderate or awkward. **b**. Classifications of neutral, moderate or awkward body angles for trunk inclination (sagittal and lateral plane) and back flexion (sagittal and lateral plane).

If the sagittal inclination of the trunk has an angle range of ≥60°, a lateral inclination of ≥20°, and a torsion of ≥20°, the trunk posture is inappropriate [23]. Beside the trunk inclinations in the sagittal and lateral plane, there are also classifications of neutral, moderate or awkward body angles for other body regions (head inclination, neck flexion and back flexion; Figures 1a + b) [21].

One-dimensional movement axes as described in ISO and DIN [29-31] norms do not correlate to real-term conditions of body posture and movement patterns during a working day. In fact, combined movements of varied degrees of freedom have to be assumed.

### Aims

The two main evaluation criteria are a general description of movement sequences and the analysis of the individual range of motions within one task. These two criteria provide an opportunity to classify the quality of movement.

Furthermore, these criteria for occupational movement quality offer the possibility to perform intra- and inter-professional comparisons.

These results may help to optimize working routine and ergonomic improvements by using a biomechanical analysis system (CUELA).

Thus, the following hypotheses for each category of junior physicians in conservative medical disciplines (intra-professional comparison) to be tested are:

1. The number of combined movements including at least two degrees of freedom exceeding the number of “simple movements” (torsion > flexion/extension; lateral-flexion).
2. Most occupational movements occur in the cervical and thoracic spine area compared to the lumbar spine area, hip or leg movements.
3. Dynamic movements are more likely to be identified than static postures. Dynamic activities have a high frequency and are performed two or more times per minute for a longer period.
4. Most upper body inclinations have a motion range of between 20° and 60°.

In addition, an ergonomic assessment for different medical disciplines of physicians (inter-professional comparison) is to be undertaken that points out varied workloads. The inter-professional hypotheses of this movement sequence analysis to be tested are:

1. Do differences exist in the execution of workloads between physicians in different medical disciplines?
2. Do different work and organization models have an effect on the range of motion as well as on the movement sequences of physicians?
3. What kinds of workload factor can be observed when comparing different medical disciplines?
4. Do specific factors exist that have a substantial influence on a physician’s movement apparatus?

## Methods

### Subjects

For this research project 21 male or female junior physicians of every conservative medical discipline will be covered. The gender ratio should largely be balanced. Data will be collected over one working shift (approx. 9 hours). This project includes junior physicians in, for example, the following disciplines: oncology, conservative medicine (general practitioner’s internist), angiology, gastroenterology, cardiology, rheumatology, geriatrics, pediatrics, child and adolescent psychiatry and psychotherapy, clinical pharmacology, neurology, emergency medicine, physical and rehabilitative medicine, psychiatry and psychotherapy, radiology, forensic medicine, radiotherapy, and transfusion medicine. In addition, the following can be covered as clinical and theoretical disciplines: occupational medicine, human genetics, hygiene and environmental medicine, microbiology, virology and infectious disease epidemiology, public health, pathology, pharmacology, and toxicology. The research project aspires to collect the largest amount of data from different medical disciplines for subsequent comparison.

As inclusion criteria the subjects should work as junior physicians in a discipline at the clinical center of the Goethe University in Frankfurt am Main, Germany. Furthermore, the absence of any posture-related disorder that might influence the execution of ordinary working tasks is required. Exclusion criteria are acute or chronic discomfort in the posture or movement apparatus. Any kind of previous treatment, such as surgery, of the musculoskeletal system should date back at least two years. The data collected serves as reference values for the work of physicians at the hospital department, so morning and afternoon shifts are equally represented. In- and exclusion criteria are evaluated in personal interviews.

This study was approved by the ethics board for research involving human subjects of the Goethe University (135/14) in Frankfurt am Main, Germany.

### Recruitment

An official letter to the senior physician or head of department heralds the planned investigation and contains the most basic information. Selection of junior physicians is on a voluntary basis after making contact.

Following their agreement to participate the physicians are informed in person about the goals and the approach of the study.

Surgeons are initially excluded due to hygiene-related concerns, especially in the operating rooms. Based on the findings of the planned research project, surgeons may be evaluated in a second step.

### Posture and motion capturing

**Figure 2:**
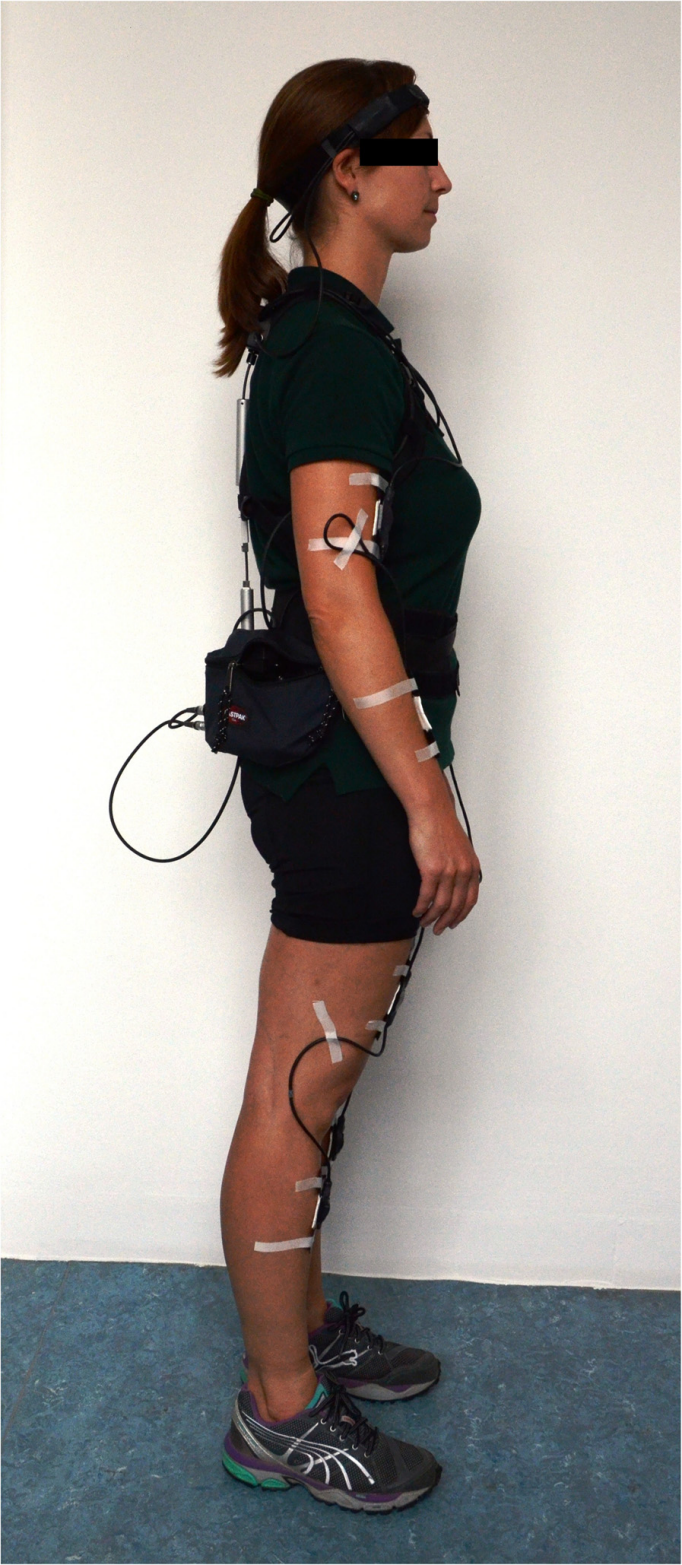
**Illustration of a complete setup of the CUELA System.**

For objective long-term measurement of loads on the musculoskeletal system the CUELA system is used (Figure 2). The basic version was developed at the IFA in 1994 [18,19]. It allows kinematic measurements of the trunk, the head and the upper and lower extremities. The system used in this study uses accelerometers (Analog Devices ADXL 103/203) and gyroscopes (muRata ENC-03R) to detect the inclination of all body segments and a digital rotary encoder to measure torsion of the spine. It is attached to the individual and can detect movement sequences and movement patterns under real conditions with a sampling rate of 50 Hz.

The angular accuracy of the sensors is better than 1° in quasi-static conditions [20,22,23,32,33].

Position and angular information provide the necessary data for reconstructing postures and movements. Since the sensor cables are attached directly to the subject, he or she can easily move in all three spatial dimensions. All data is stored in a data storage unit with a flash memory card that is attached to the subject. The storage unit containing a rechargeable battery can be read out on a PC for further data editing and analysis.

The IFA [18] has validated in its laboratory the repeatability and long-term stability of the system. The results show only slight deviations. Furthermore, comparative measurements of the CUELA system and an optical motion capture system (VICON, Oxford, UK) were conducted to investigate whether attaching the sensors to working clothes led to imprecision. The mean deviation of the systems compared was in the range of <4% for the trunk angle in the sagittal plane and for the hip and knee joint angles. Comparison of torsion and lateral flexion angles of the trunk showed greater deviations. One reason for these differences might be the angle calculation from the absolute marker coordinates of the Vicon system. The deviation range was confirmed by Morlock et al. [34].

### Measurement protocol

All physicians are instructed to go about their ordinary daily working activities. They are told not to increase or decrease the frequency or the range of motion of any activity during recording. In former investigations an accompanying video was used that recorded the working tasks and allowed precise subsequent assignment of the measurement data to each task. This provided inspection and optimal further data analysis. For data protection reasons a video recording is impossible in the investigations planned. Instead of recording the tasks manually, a computer-based observation system is used. Mache et al. [6-14] evaluated this system and conducted several investigations in the medical health sector. For this purpose special software was developed. This time recording software is implemented in an ultra-mobile PC that is a handheld laptop with a pressure-sensitive screen and translates all job tasks into task categories.

At the beginning and the end of the measurement procedure each subject has to stand in a habitual upright posture to initiate the measurement. Every individual habitual posture is defined for each subject. Comparison between the initial and the final measurement of the upright posture guarantees that the sensors have not been displaced during the measurement period. One measurement sequence lasts approximately 7 to 9 hours.

**Figure 3:**
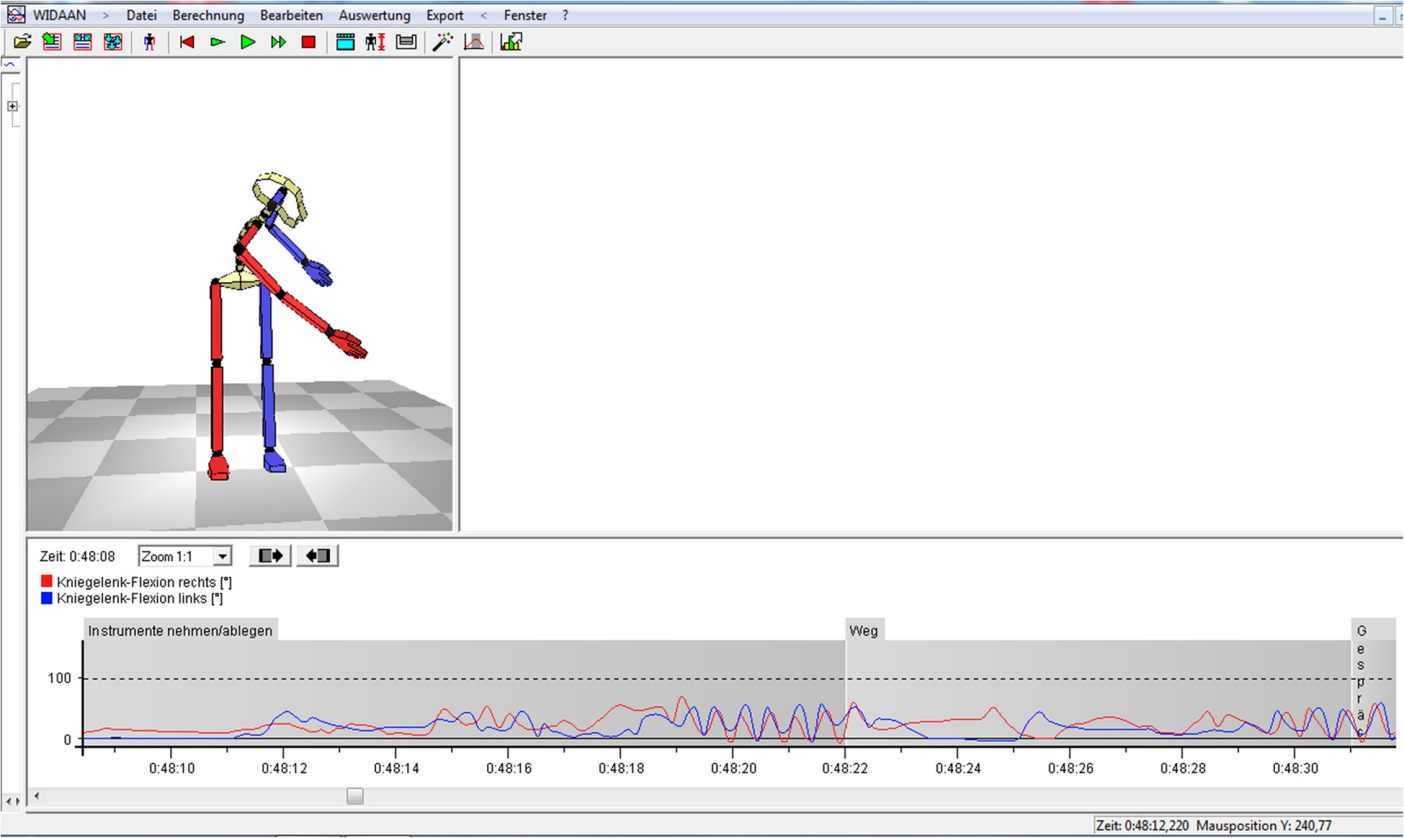
**Illustration of the CUELA-software interface.**

Calculation of statistical parameters and graphical models is easily carried out by the specially developed CUELA software. An animated computer figure includes all previously recorded data and synchronizes the data of the CUELA system and the working tasks observed (Figure 3).

### Evaluation criteria

First, an adequate categorization system should be developed to enable exemplary description of movements and body posture, including subsequent degree classification of tasks, movement categories, and repetitive movement patterns, especially in the trunk region. These categorized movement sequences are the most important basis for this analysis and further research on human movements. An ergonomic evaluation is therefore carried out in addition to the posture evaluation. Before analyzing these parameters, basic calculations are necessary (in degree).

The movements of the upper body can be analyzed on four planes by all sensors:

- Sagittal direction (flexion/extension)
- Lateral movements come from the central axis (laterotrusion)
- Torsion
- Bending (difference of two close sensors, i.e. the thoracic and the lumbar sensor).

In general, the following body angles as well as their degrees of freedom are recorded by the CUELA system and are listed as follows (Table 1):

**Table 1:** **Joints/body regions and their degree of freedom measured by the applied sensors of the CUELA system used in this study**

**Joint/body region**	**Degree of freedom**
Head	Sagittal and lateral inclination
Thoracic spine	Sagittal and lateral inclination at the level of TH4
Lumbar spine	Sagittal and lateral inclination at the L5 level
Thoracic and lumbar spine	Torsion between thoracic and lumbar spine
Hip and knee joints	Flexion/extension
Shoulder joint	Flexion/extension
Elbow	Flexion/extension

The trunk inclination angle was calculated from the average Th4 and L5 sagittal inclination angles. The trunk flexion angle was defined as the angular difference between the Th4 and L5 sagittal inclination. The trunk lateral flexion angle was defined as the angular difference between the Th4 and L5 lateral inclination.

The aim of the evaluation parameter is the summation of the individual time elements of the adopted postures and activities over the entire work shift. Upper body posture in which the subject exceeds the normal range will also be added in time.

### Statistical data analysis

Subsequent to the descriptive statistics, adequate statistical tests in accordance with the respective data and depending on the appropriate hypothesis are conducted.

The primary aim is to determine the relative frequency of combined movements including at least two degrees of freedom with the corresponding 95% confidence interval compared to the number of all movements. A comparison between the absolute frequencies of movements in the cervical and thoracic spine area and those in the lumbar spine area and to hip or leg movements is conducted. Furthermore, an analysis of the difference in the frequency of dynamic and static movements is performed. The difference between the frequencies will be analyzed with a two-sided exact binomial test with a significance level of α = 5%. In a second analysis we will estimate two-sided 95% confidence intervals for the difference in the frequency of those movements.

The completed analysis will be registered, including all task aspects such as the kind of work activity (static or dynamic), duration, frequency, intensity and range of motion of the tasks, repetition of angular velocity and frequency as well as interruptions (frequency, duration, type).

Statistical analyses are performed using BiAS 10.8 (Epsilon Verlag, Norderstedt/Germany).

## Discussion

There are numerous approaches to assessing work hazards in the healthcare professions [15,27,35,36]. Up to now only data for real-time observation studies on working activity or physicians’ workload in the clinical field has existed.

This study is the first computer-based investigation of the musculoskeletal system by means of the CUELA system that offers the possibility to evaluate the quality of movement sequences of junior physicians’ work in different conservative medical disciplines. Biomechanical parameters on loads of the musculoskeletal system as collected by the CUELA system enable already known real-time descriptions of work activities as published by Mache et al. [5-14] to be extended. Consequently, it is possible to record the activities undertaken in a working day and in particular the quality of the movements over a working day. This analysis provides information about the frequency of movements and the range of junior physicians’ movements working in inpatient care during a work shift. In this way awkward posture, static posture, highly dynamic work, repetitive work, and acting forces are analyzed biomechanically and conclusions about frequent/infrequent movements or rather movement sequences in their intensity can be drawn.

Medical doctors are chosen as subjects because the physician’s health and physical exertion are mostly neglected although complaints of their musculoskeletal system should not be neglected. Investigations on the movement apparatus of physicians do not exist so far. In addition, physicians have an important position in the German public and occupational health system. They guarantee the productivity of society by enabling people in a country to regain and recover their health. That is why physicians’ health and hence their productivity is the main focus of this study design. In particular, physicians in different medical disciplines are the first subjects covered. But nonetheless, the same analysis with regard to nurses and physicians’ assistants and dental assistants is necessary and useful to detect a consistent analysis of all professions in the medical care system, as these professions have been neglected so far as well as physicians.

### Limitations

Given the expected difficulties of these planned investigations it must be borne in mind that the Hawthorne effect may have an impact on the expected results, especially in this study design. It is a possible explanation for the distortion of results in non-blind intervention studies and involves behavioral changes due to awareness of being observed. This combines the active compliance of the test person as well as the presumed wishes of researchers [37]. In contrast, a distortion of the results by the investigator is not likely to have an influence on this analysis of the restricted results in terms of pure numbers and defined evaluation criteria [38].

Logical consequences on the basis of these results are the formulation of adequate assessments of job tasks as well as the creation of ergonomic design, prevention, and rehabilitation programs.

Consequently, a central and a long-term goal is to develop intra-professional and inter-professional possibilities of improvement to optimize physicians’ work tasks in terms of reducing awkward postures and movements by answering the hypotheses stated above. Periods of high workload that may lead to posture related disorders and might have an impact on the quantity and quality of essential movement sequences can thus be avoided. As a result the physician’s physical health will be increased and, as an indirect effect, better patient care can be assumed. This study can thus stimulate an overall improvement in physicians’ subjective physical wellbeing in Germany.
